# Evolutionary trade‐offs of insecticide resistance — The fitness costs associated with target‐site mutations in the nAChR of *Drosophila melanogaster*

**DOI:** 10.1111/mec.15503

**Published:** 2020-06-22

**Authors:** Rafael A. Homem, Bliss Buttery, Ewan Richardson, Yao Tan, Linda M. Field, Martin S. Williamson, T. G. Emyr Davies

**Affiliations:** ^1^ Rothamsted Research Biointeractions and Crop Protection Department Harpenden, Hertfordshire UK; ^2^ Research Centre for Grassland Entomology Inner Mongolian Agricultural University Hohhot China

**Keywords:** CRISPR, insecticide resistance, nAChR_α6, nAChR_β1, neonicotinoids, nicotinic acetylcholine receptor, R81T, spinosad

## Abstract

The evolution of resistance to drugs and pesticides poses a major threat to human health and food security. Neonicotinoids are highly effective insecticides used to control agricultural pests. They target the insect nicotinic acetylcholine receptor and mutations of the receptor that confer resistance have been slow to develop, with only one field‐evolved mutation being reported to date. This is an arginine‐to‐threonine substitution at position 81 of the nAChR_β1 subunit in neonicotinoid‐resistant aphids. To validate the role of R81T in neonicotinoid resistance and to test whether it may confer any significant fitness costs to insects, CRISPR/Cas9 was used to introduce an analogous mutation in the genome of *Drosophila melanogaster*. Flies carrying R81T showed an increased tolerance (resistance) to neonicotinoid insecticides, accompanied by a significant reduction in fitness. In comparison, flies carrying a deletion of the whole nAChR_α6 subunit, the target site of spinosyns, showed an increased tolerance to this class of insecticides but presented almost no fitness deficits.

## 1 INTRODUCTION

The assumption that resistance to xenobiotics is associated with fitness costs forms the basis of many resistance management strategies in medicine (antimicrobial resistance) (Melnyk, Wong, & Kassen, 2015), agriculture and disease vector control (pesticide resistance) (Coyne, 1951; Georghiou, 1994). Such strategies rely on the alternation of antimicrobial (antibiotic) or pesticide compounds with differing modes of action and presuppose that the accompanying fitness costs will select against resistance to one compound while a second one is in use. Despite playing a central role in the formulation of resistance management strategies, fitness costs associated with specific instances of resistance are still understudied, particularly in the pesticide resistance field (Cloyd, 2010). The aim of the present study was to investigate whether specific resistance mutations, when introduced into the *D. melanogaster* nicotinic acetylcholine receptor (nAChR), would adversely affect the fitness of flies when they were not under insecticide selection pressure. It is generally perceived that in the absence of insecticides, susceptible individuals retain reproductive or other advantages, and therefore, the proportion of resistant individuals in a population would tend to decrease over time.

nAChRs are ligand‐gated ion channels that mediate the fast action of acetylcholine (ACh) in synaptic cholinergic transmissions, thus regulating processes such as cell excitability and neuronal integration (Cecchini, 2015). In insects, nAChRs are restricted to the nervous system, where they are present at high densities and are the targets of several classes of insecticides including neonicotinoids, spinosyns, sulfoxamines and butenolides (Casida, 2018), which combined account for almost 22% of the insecticide global market (Crossthwaite et al., 2017).

Neonicotinoids are highly selective agonists of insect nAChRs and are very effective against a range of important insect pests, particularly piercing‐sucking pests such as aphids, whiteflies and plant hoppers (Jeschke, Nauen, Schindler, & Elbert, 2011). The success of this class of insecticide can be attributed to their versatility in terms of delivery methods, their high affinity to insect nAChRs and their relatively low toxicity to vertebrates (Casida, 2018). Despite their widespread use, neonicotinoids have proven to be remarkably resilient to the development of resistance (Bass, Denholm, Williamson, & Nauen, 2015). In fact, the first field‐evolved target‐site mutation associated with resistance to neonicotinoids was reported only in 2011 (Bass et al., 2011), 20 years after they were first introduced to the market. This mutation, which leads to an arginine‐to‐threonine substitution at position 81 (R81T) of the nAChR_β1 subunit, was first found in a clone (FRC) of the peach‐potato aphid, *Myzus persicae,* isolated from a peach orchard in the south of France (Bass et al., 2011) and later in field populations of the cotton aphid, *Aphis gossypii,* sampled across East Asia (Koo, An, Park, Kim, & Kim, 2014; Shi et al., 2012). To date, the R81T mutation has only been found in these two species, suggesting that it might carry strong fitness costs in other insects.

Spinosyns are natural, broad‐spectrum insecticides produced by the microorganism *Saccharopolyspora spinosa* under aerobic fermentation (Sparks et al., 1998). They act primarily as allosteric agonists of the nAChR, causing involuntary neuronal excitation, muscle contraction, paralysis and death (Salgado & Sparks, 2005). The nAChR_α6 subunit has been proposed as the main target of this class of insecticides and this is supported by the finding that mutations in the *nAChR_α6* gene of various insects cause insensitivity to spinosad, an insecticide comprising two spinosyns (A and D) (Baxter et al., 2010; Berger et al., 2016; Hsu et al., 2012; Perry, McKenzie, & Batterham, 2007; Puinean, Elias, et al., 2013; Rinkevich, Chen, Shelton, & Scott, 2010; Silva et al., 2016). Spinosad was first introduced to the market in 1997, and despite efforts to try to mitigate the evolution of resistance, it did not take very long before the first control failures were reported. In the island of Hawaii, it took just over 2.5 years for spinosad to become ineffective against the diamondback moth, *Plutella xylostella* ( Zhao et al., 2002). An attempt to mitigate against the spread of spinosad resistance led to a voluntary withdrawal from the Hawaiian market whilst newly registered insecticides (emamectin benzoate and indoxacarb) were applied. These efforts were partially successful, with some *P. xylostella* populations showing a moderate increase in susceptibility to spinosad. However, once spinosad was re‐introduced, resistance reappeared very rapidly (Zhao et al., 2006) and was attributed to frameshift mutations leading to the truncation of the nAChR_α6 subunit (Baxter et al., 2010; Rinkevich et al., 2010). It can be speculated that the failure to restore susceptibility in field populations of *P. xylostella* might be due to the lack of fitness costs associated with the absence of the nAChR_α6 subunit in the receptor.

To test the hypothesis that target‐site resistance to spinosyns carries a much lower fitness cost than does the target‐site resistance to neonicotinoids, CRISPR/Cas9 was used to generate two genome‐edited *D. melanogaster* strains. One strain had a deletion of the *nAChR_α6* gene, and the other had the R81T substitution in the nAChR_β1 subunit. Insecticide bioassays were used to test whether the modified strains exhibited the expected insecticide‐resistant phenotypes, and fecundity, fertility, larval crawling, negative geotaxis (climbing performance) and longevity (lifespan) assays were used to test whether either or both of the mutations conferred a fitness cost.

## 2 MATERIALS AND METHODS

### 2.1 *D. melanogaster* strains

Most strains were kept on standard fly food (Bloomington formulation) at 19°C, 12:12‐hr photoperiod. Due to the low fecundity of the strain, the R81T homozygous stock was kept at 25°C, 12:12‐hr photoperiod and 65% RH. In addition, a strain carrying the R81T mutations over a balanced chromosome (R81T/ TM6B, Tb^1^, Hu^1^) was kept at 19°C, 12:12‐hr photoperiod. The Cas9 strain, used as the control strain, has been described elsewhere (Zimmer et al., 2016). This strain is deficient for the DNA *ligase 4* gene and expresses *Cas9* under the control of the *vasa* germline‐specific regulatory elements (genotype w1118, Lig4169; PBac{y[+mDint2]=vas‐Cas9}VK00027). All heterozygous strains originated from crosses using the control strain as mothers and the gene‐edited flies as fathers. The balancer strain (genotype w*; Sp/CyO; Sb/TM6B, Tb^1^, Hu^1^) was obtained from Professor David Finnegan, University of Edinburgh, UK.

### 2.2 CRISPR design and plasmid construction

Two gRNA expression plasmids were generated—one to “knock‐in” the R81T mutations in the *nAchR_β1* gene and the other to “knock‐out” the *nAchR_*α6 gene. Both constructs used the pCFD4‐U6:1_U6:3 tandem gRNA plasmid (addgene #49411) as a backbone. This plasmid expresses two gRNAs under the control of the *D. melanogaster* U6:1 and U6:3 promoters (Port, Chen, Lee, & Bullock, 2014). The online platform CHOPCHOP (accessed at http://chopchop.cbu.uib.no/) (Labun, Montague, Gagnon, Thyme, & Valen, 2016) was used to screen for suitable gRNA target sites in the latest (release 6) reference genome assembly of *D. melanogaster* (Dos Santos et al., 2015). Both on‐target activity and off‐target interactions were taken into consideration when choosing the two most suitable gRNAs for each gene. All gRNA target sites were validated by PCR amplification and sequencing. Primers used to amplify the fragments containing the gRNA target sites are listed in Table S1. The gRNAs were cloned into the pCFD4‐U6:1_U6:3 tandem gRNA plasmid as described elsewhere (Port et al., 2014). Briefly, primers were designed to integrate two gRNA sequences into a single PCR product using pCFD4‐U6:1_U6:3 tandem gRNAs as template in the PCR. The PCR products were then cloned by homology‐directed cloning (Gibson Assembly® Cloning Kit—NEB) into the backbone of the pCFD4‐U6:1_U6:3 tandem gRNA plasmid previously digested with BbsI. These and all other plasmids were propagated in *E. coli* (NEB^®^ 5‐alpha) and purified using the GeneJET Plasmid Miniprep Kit (Thermo Fisher Scientific) according to the manufacturer's instructions.

The R81T donor plasmid was synthesized *in vitro*. A single 1004‐bp homology template containing the desired point mutations was designed using Geneious R8 (Biomatters, Auckland, New Zealand) and sent to GeneArt® Gene Synthesis Service (Thermo Fisher Scientific) for synthesis. This fragment contained nine nucleotide changes; seven synonymous mutations introduced in the gRNA target sites to prevent Cas9‐mediated DNA breaks after integration and two conferring the R81T substitution and creating a HpaI restriction site at that position. The final construct provided by Thermo Fisher Scientific used their standard pMA cloning plasmid as a backbone.

The α*6* (A6) donor plasmid was generated by standard restriction cloning protocols. Two 1,000‐bp fragments with homology to upstream (5’) and downstream (3’) regions flanking the *nAChR_*α*6* gene were amplified from the genome of the Cas9 strain (control flies) by PCR using Phusion High‐Fidelity DNA polymerase (NEB) (see Table S1 for primer sequences). PCR fragments were purified and cloned into pJET1.2/blunt cloning vector using the CloneJET PCR Cloning Kit (Thermo Fisher Scientific). Fragments were excised from subcloned plasmids using EcoRI/SphI for the 5’ fragment and BglII/XhoI for the 3’ fragment and T4 ligated into the multiple cloning sites of a modified pDsRed‐attP plasmid (addgene #51019) called pDsRed‐2attP. This modified plasmid contains a second inverted attP sequence downstream of the DsRed marker and had been generated using primers: attP2_BglII_F and attP2_SpeI_R (Table S1) and standard digestion–ligation cloning protocols. The final A6 donor plasmid (pDsRed‐2attP‐A6_HAs) contained the 3xP3‐DsRed marker flanked by two inverted attP sites, flanked by two 1000‐bp homology arms.

### 2.3 Embryo microinjections and molecular screening

Constructs were microinjected into nondecoronated syncytial blastoderm embryos of the Cas9 strain of *D. melanogaster* using an inverted microscope (Eclipse TieU, Nikon, Japan) equipped with a 10x/0.25 lens, 10x/22 eyepiece and fluorescence illumination. Injection mixtures (0.5x phosphate buffer pH 6.8 (0.05 mM sodium phosphate, 2.5 mM KCl), 100 ng/µl of a pCFD4‐U6:1_U6:3 tandem gRNA plasmid, 200 ng/µl of a donor plasmid and 200 mg/L fluorescein sodium salt) were delivered by a FemtoJet express microinjector (Eppendorf, Hamburg, Germany) controlled by a motorized TransferMan NK2 micromanipulator (Eppendorf, Hamburg, Germany). Injection needles were made of quartz and were prepared using a P‐2000 micropipette puller (Sutter Instrument Co, Novato, USA).

For the generation of Δα6 flies, microinjection survivors were backcrossed to the Cas9 strain in a three‐to‐one (Cas9 strain to microinjection survivor) ratio per vial and kept at 25°C, 12:12‐hr photoperiod 65% RH. The F1 progeny were screened for the expression of DsRed in the eyes and ocelli. DsRed‐positive flies were intercrossed, and F2 flies were screened for a stronger expression of DsRed (homozygous flies carrying two copies of the transgene). F2‐positive flies were then intercrossed to generate homozygous stocks. Screenings were carried out on freshly emerged flies under CO_2_ anaesthesia using a dissecting microscope (Leica MZ10 F) equipped with an epifluorescence light source and an ET‐DsRed filter system. Knock‐in of the transgene was validated by PCR and sequencing using primer pairs (Table S1) that could only generate a fragment when the integration had taken place at the expected genomic position (illustrated in Figure 2b). A6 knock‐out was also validated by RT–PCR. Total RNA was extracted from homozygous Δα6 and control flies using TRIzol (Invitrogen, CA) according to the manufacturer's protocol. cDNA was synthesized using SuperScript™ III Reverse Transcriptase (Thermo Fisher Scientific) following the manufacturer's instructions. 3 µl of 1/10 cDNA dilutions was used in PCRs with primers (A6_RT–PCR_F/R) designed to amplify a 416‐bp fragment including part of exon 11, exon 12 and part of exon 13. PCR products were verified by electrophoresis in 1% w/v agarose gel. Primers to amplify a fragment of the *RpL32* housekeeping gene were used in control reactions.

A simplified crossing scheme used to generate homozygous R81T flies is shown in Figure S1. First, microinjection survivors were crossed to the double balancer strain in a three‐to‐one (balancer strain to microinjection survivor) ratio per vial and kept at 25°C, 12:12‐hr photoperiod 65% RH. After a week, pools of around 20 pupae were sampled and genotyped by PCR and sequencing. Genomic DNA from these samples was isolated using DNAzol™ Reagent (Thermo Fisher Scientific) following the manufacturer's protocol and used as template in PCRs using the primers B1_Seq_HA_Right_F/R (Figure S1). The 600‐bp fragments amplified from the reactions were gel‐purified and sequenced. Samples were considered positives when sequencing traces showed double peaks at expected positions. This led to the identification of vials containing positive F1 flies. From these vials, all TbHu F1 flies (males and virgins females) were collected and subsequently crossed to the double balancer strain in a three‐to‐one ratio per vial. After F2 larvae were seen in the vials, potentially edited F1 parents were collected and individuals were genotyped by PCR and sequencing as described above. Non‐Sb but TbHu F2 adults (males and virgin females) generated from positive F1 parents were selected and intercrossed. The final R81T homozygous stocks were generated by selecting and intercrossing non‐TbHu F3 flies. Stocks were validated by PCR, sequencing and digestion with the HpaI restriction enzyme.

### 2.4 Insecticide bioassays

Analytical grade acetamiprid, flupyradifurone, imidacloprid and spinosad (Sigma‐Aldrich, St. Louis, MO, USA) were resuspended in 100% acetone and serial‐diluted in 50% v/v acetone/ 50% water solution. A commercial formulation of sulfoxaflor (Isoclast™ active—Dow AgroSciences) was serial‐diluted in 100% water. 100 µl of each dilution (fivefold dilutions) was added to the surface of 3ml of 2% w/v agar containing 1.2% w/v sucrose and 0.4% v/v glacial acetic acid (Sigma‐Aldrich, St. Louis, MO, USA) in 5x95mm vials one day prior to use, to allow for the complete distribution of the insecticide and evaporation of the solution.

Young adult female flies (4–8 days old) were used in the contact/feeding bioassays to assess the impact of each mutation on insecticide resistance. Heterozygous flies were generated from crosses using the control strain as the mother. 20 flies of each genotype were transferred to insecticide‐treated vials, 5 vials were prepared for each concentration and at least 5 concentrations were tested per bioassay. Bioassays were kept at 25°C, 60% RH and 12:12‐hr photoperiod. After 48h, the number of dead flies was counted, and the lethal concentrations necessary to kill 50% of the flies (LC50) were calculated by probit analysis using Genstat version 18 (VSN International). The mode of inheritance was calculated by applying the respective LC values (Stone, 1968). Nonlinear log dose–response curves were generated in GraphPad Prism 6.0 (GraphPad Software Inc., La Jolla, CA, USA).

### 2.5 Fecundity and fertility assays

To calculate fecundity, the number of eggs laid per female over 24 hr was assessed. Around 70 to 100 freshly emerged couples of each genotype were transferred to 25x95mm vials containing 5 ml of red‐coloured fly food (Bloomington formulation plus food dye) at 10 couples per vial. Flies were kept at 20°C and transferred to fresh vials without anaesthesia after three days. 24 hr later, flies were removed from the vials and the number of eggs was recorded. To calculate fertility, the proportion of pupae to eggs was calculated using the vials from the fecundity assay. To calculate larval and pupal survivals, crosses containing around 150 couples were allowed to lay eggs on molasses/agar plates (2% agar, 15% molasses, 0.8% propionic acid) supplemented with yeast paste for a period of 24 hr. Molasses plates were collected and used to randomly select 100 L1 larvae of each genotype, which were transferred to food vials at 10 larvae per vial. The number of pupae and adults in these vials was recorded and used to calculate the pupation and eclosion rates.

### 2.6 Crawling assays

Six‐well plates filled with molasses/agar were prepared as arenas for crawling speed assays. Around 20 second instar larvae of each genotype were transferred to the wells of the plate. To stimulate crawling, a drop of yeast solution was placed in the middle of the wells just before video recordings started. Videos were recorded at 25 frames per second for around 60 s using a Sony HandyCam. Video analyses were done using the Ctrax fly tracker software (Branson, Robie, Bender, Perona, & Dickinson, 2009) (Caltech). The average speeds were calculated in RStudio (RStudio Team, 2020) using the x, y coordinates over time.

### 2.7 Climbing assay

The climbing assay was performed with the use of an automated fly‐climbing system adapted from a previously described set‐up (Willenbrink et al., 2016). The system employs a two‐storey acrylic tube rack (measuring 550 × 400 × 50 mm) capable of holding 20 standard Drosophila vials. The rack rests on a horizontal camshaft, with asymmetrical cams that cause the rack to rise and fall within a 4mm travel as the shaft rotates. The shaft itself is driven by a Marelli Motori MAA 63MB6 electric motor, at a rate of approx. 200 rpm, resulting in a violent and consistent shaking of the vials. The rack is marked with a horizontal line, at a height of 6cm from the base of each vial, in order to assess fly‐climbing ability.

Flies used for the assay were kept on standard fly food at 20°C and transferred (by tapping, without the use of CO_2_) to empty vials and loaded into the automated climbing system. The climbing assay was carried out at 20°C and involved 5 s of vial shaking; 8 s for climbing; image capture; 45 s of resting, repeated 13 times during a single experiment. No data were collected during repeats 1–3 to allow for habituation before data collection. Images were captured with a Canon EFS digital camera with an 18‐ to 55‐mm lens, positioned on a tripod at a height level with the centre of the climbing system. Captured images were scored manually to determine the number of flies above the 6cm line in each vial and the score for each vial averaged over the 10 data points.

### 2.8 Longevity assay

To measure the longevity of mated flies, around 120 freshly emerged couples of each genotype were collected and placed in food vials. After three days, males and females were separated and transferred to fresh vials in groups of 10 flies per vial. Flies were kept at 25°C, 12:12‐hr photoperiod 65% RH for the entire experiment. Deaths were scored every day, and survivors were transferred to fresh food vials every three to four days.

### 2.9 Statistical analysis

All statistical analyses used Genstat (18th Edition, VSN International). Insecticide lethal concentrations (LC50s) were calculated by probit analysis, and LC50s were considered significantly different when 95% confidence intervals (CIs) did not overlap. Data generated from the fecundity, fertility, climbing and crawling assays were analysed using one‐way ANOVA followed by the Bonferroni post hoc test. Larvae pupation and pupae eclosion rates were analysed using the Kruskal–Wallis test. Lifespan data were analysed using the log‐rank (Mantel–Cox) test against the lifespan of the control.

## 3 RESULTS

### 3.1 Genetically engineering the nAChR of *D. melanogaster*

**FIGURE 1:**
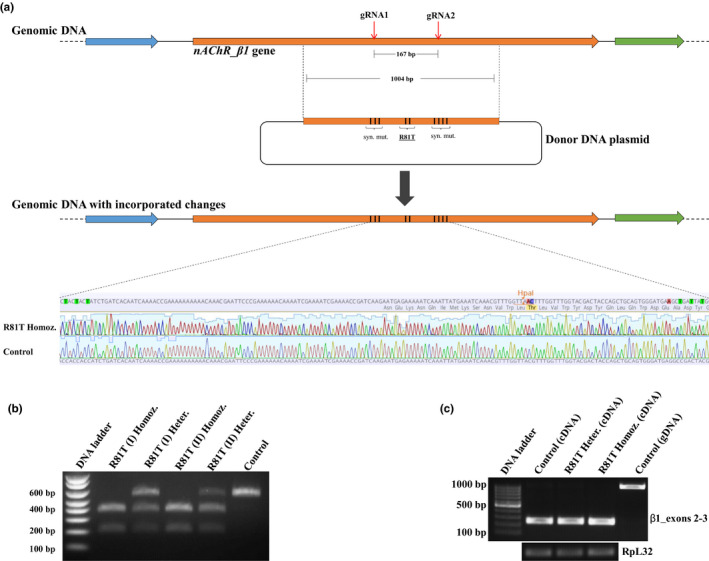
**CRISPR‐mediated knock‐in of gene mutations leading to a R81T substitution in the nAChR_β1 subunit.** **(a)** Genomic region encompassing the nAChR_β1 gene before and after R81T knock‐in. Red arrows indicate gRNA target sites. The donor plasmid carrying a 1004‐bp homology template fragment was used to introduce the desired mutations through CRISPR‐induced homology‐directed repair. Successful integration was confirmed by DNA sequencing. Nucleotide and amino acid changes are highlighted above the sequencing traces comparing control and R81T homozygous flies. **(b)** HpaI digestion of PCR products amplified from the genome of control, R81T heterozygous and R81T homozygous flies. (I) and (II) correspond to two independently generated R81T strains. Control flies produced a single, nondigested 600‐bp band following gel electrophoresis. Homozygous strains produced two smaller bands (400 and 200 bp) corresponding to a complete digestion of the PCR product. Heterozygous flies (the progeny of crosses between R81T homozygous and control flies) produced a mixture of the two with three bands being present on the gel. **(c)** RT*–*PCR analysis of the R81T region (exons 2 and 3 of *nAChR_β1* gene) from control, R81T heterozygous and R81T homozygous flies. The *RpL32* gene was used as a housekeeping control for standardizing expression levels. Genomic DNA and complementary DNA are abbreviated as gDNA and cDNA, respectively

CRISPR/Cas9 was used to edit *D. melanogaster nAChR* genes and generate two fly strains. For the first strain, a precision knock‐in strategy, using two gRNAs (Table S1) and a homology template construct (donor plasmid), introduced two nonsynonymous and seven synonymous point mutations within the *nAChR_β1* gene. The seven synonymous mutations were deliberately sited within the gRNA‐binding sites (three in one and four in the other) to prevent the re‐occurrence of Cas9‐induced double‐strand breaks at those positions once homology‐directed repair took place. The two nonsynonymous mutations were designed to give the replacement of an arginine by a threonine at position 81 and created a *HpaI* restriction site at that position. Donor and gRNA plasmids were co‐injected into syncytial blastoderm embryos of a transgenic strain that expresses the *SpCas9* gene in the germline cells under the control of the *vasa* promoter. Two out of 40 injected survivors transmitted the mutations to their offspring (Figure 1a). The detailed screening strategy used to identify the genome‐edited flies and to generate R81T homozygous strains is shown in Figure S1 (and described in Material and Methods). Further validation, using restriction digests, confirmed the presence of the *HpaI* site in the target region of engineered flies (Figure 1b). The introduced mutations had no effect on the levels of transcription or splicing of the *nAChR_β1* mRNA, as RT–PCR amplification of a fragment that included parts of exons 2 and 3 and covered all the introduced mutations showed that neither the intensity nor the size of the transcripts changed in the engineered flies compared with control flies (Figure 1c).

**FIGURE 2:**
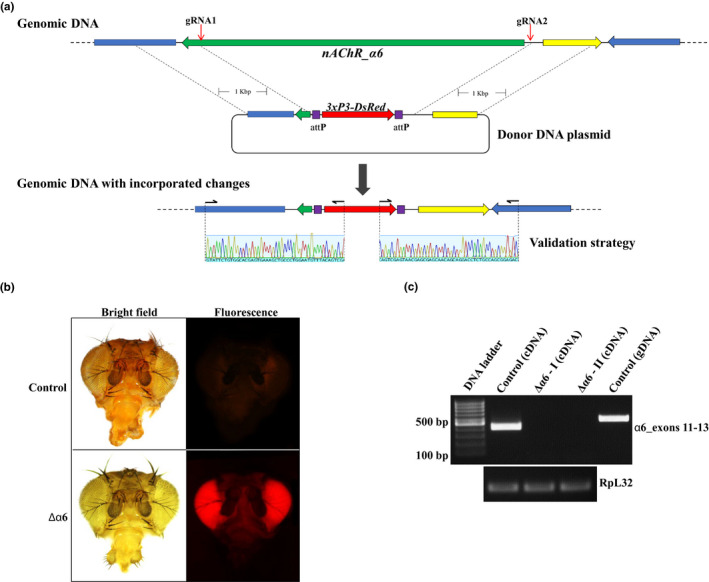
**CRISPR‐mediated knock‐out of the *nAChR_α6* gene.** **(a)** Genomic region encompassing the *nAChR_α6* gene before and after CRISPR‐mediated knock‐out. Red arrows indicate gRNA target sites. The donor plasmid carrying the 3xP3‐DsRed marker surrounded by two 1‐Kbp homology arms was used to replace the gene by the marker through CRISPR‐induced homology‐directed repair. Primers used to validate the knock‐in/knock‐out event are shown above sequencing traces. Sequencing traces shown in this figure are representative only. **(b)** Heads of a control fly and a nAChR_α*6* knock‐out fly under bright field and epifluorescence light. nAChR_α*6* knock‐out flies express the DsRed marker in the eyes under the control of the 3xP3 promoter. **(c)** RT*–*PCR analysis of a region of the *nAChR_α6* gene (exons 11 to 13) from control and two independently generated α*6* knock‐out strains (Δα*6* I and II). The *RpL32* gene was used as a housekeeping control for the expression levels. gDNA and cDNA stand for genomic and complementary DNA, respectively

For the second edited fly strain, a knock‐out of the *nAChR_*α*6* gene (Δα6) was achieved by employing a marker knock‐in/gene knock‐out approach. Two gRNAs, designed to target both ends of the gene, and a donor plasmid carrying two 1‐kb homology arms surrounding the 3xP3‐DsRed marker were used to induce the replacement of the full gene by the fluorescent marker (Figure 2a). Flies carrying the deletion were identified by screening the F1 generation for the expression of DsRed in the eyes of adult flies (Figure 2b). F1 heterozygotes were then intercrossed and F2 homozygotes were identified based on the intensity of DsRed, as the intensity of the fluorescence is higher in homozygous strains carrying two copies of the marker. RT–PCRs confirmed the complete knock‐out of the gene in the engineered flies as no transcripts could be detected in two independently generated knock‐out strains (Figure 2c).

### 3.2 Mutations in the nAChR of *D. melanogaster* alter the levels of resistance to multiple insecticides

**FIGURE 3:**
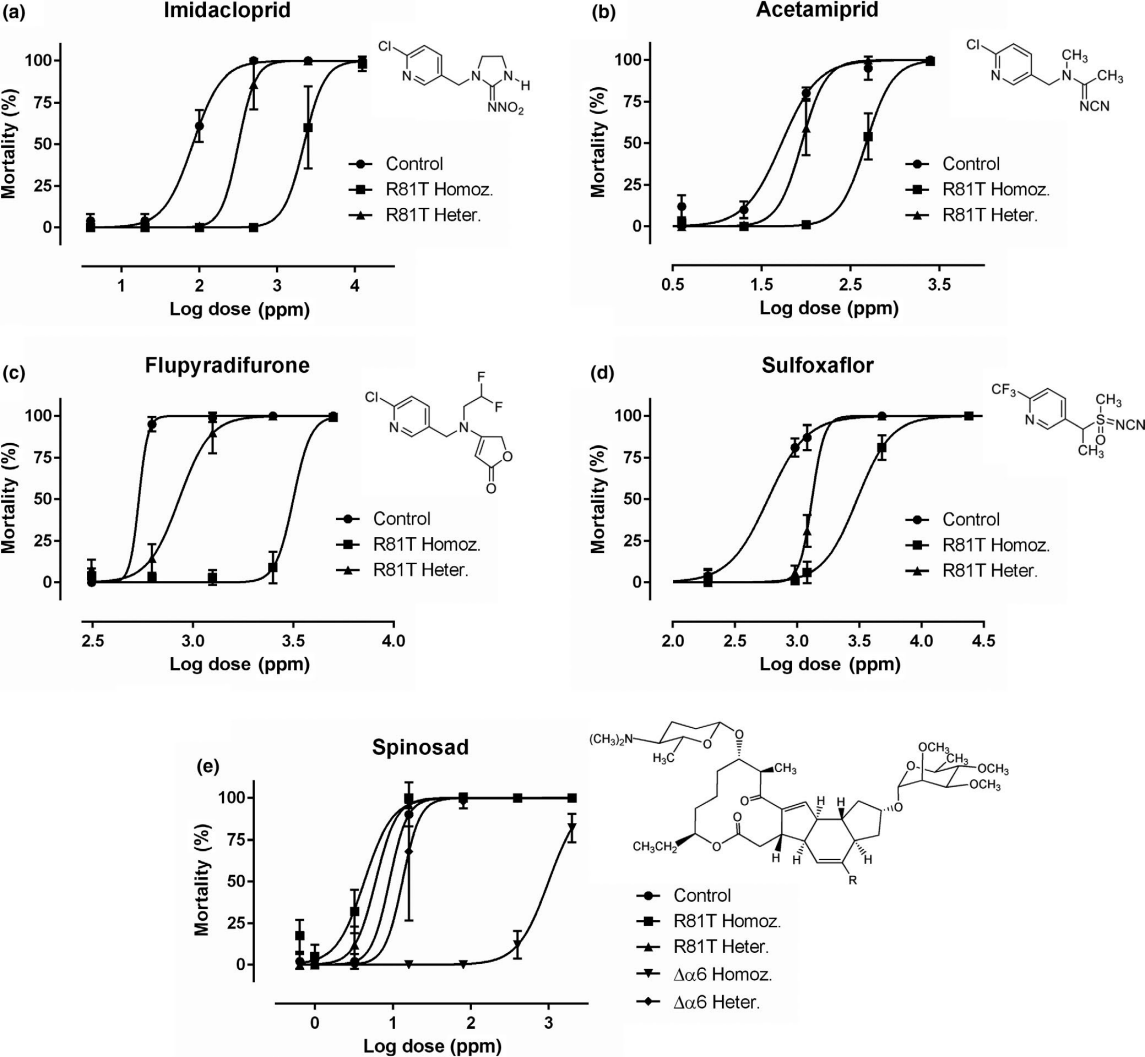
**Nonlinear log dose–response curves for five agonists of the nAChR.** Mortality of control and genome‐edited female flies after 48 hr exposure to increasing doses of **(a)** imidacloprid **(b)** acetamiprid **(c)** flupyradifurone **(d)** sulfoxaflor and **(e)** spinosad. Error bars represent standard deviations

To confirm the role of the mutations in resistance to insecticides, genome‐edited flies from the two strains were tested in dose–response mortality assays using five agonists of the nAChR: imidacloprid, acetamiprid, sulfoxaflor, flupyradifurone and spinosad (Table 1, Figure 3). The first four insecticides bind at the α‐β interface of the receptor (Casida, 2018), whilst spinosad binds to the C‐terminal region of the α6 subunit (Somers, Nguyen, Lumb, Batterham, & Perry, 2015). All heterozygous flies used originated from crosses using the control as mothers and the genome‐edited flies as fathers.

**TABLE 1:** Log dose probit mortality data

Insecticide	Strain	LC50 (mg/L)	95% CI	Resistance ratio	Dominance
**Imidacloprid**	Control	69.9	53.4–91.5	–	–
	R81T Heter.	300.1	256.6–341.6	4.3	–
	R81T Homoz.	2,276	1,851–2,791	32.6	−0.847
**Acetamiprid**	Control	66.4	45.6–88.8	–	–
	R81T Heter.	86.2	76.7–96.3	1.3	–
	R81T Homoz.	433	305.6–618	6.5	−0.892
**Sulfoxaflor**	Control	571	521.5–620	–	–
	R81T Heter.	1,332	1,282–1,411	2.3	–
	R81T Homoz.	2,930	2,382–3,736	5.1	−0.355
**Flupyradifurone**	Control	479.1	442–514.1	–	–
	R81T Heter.	892	802–977	1.9	–
	R81T Homoz.	3,290	3,015–3,633	6.9	−0.706
**Spinosad**	Control	7.7	6.0–9.7	–	–
	R81T Heter.	6.1	5.4–7.0	0.8	–
	R81T Homoz.	3.1	2.2–4.3	0.4	0.304
	Δα6 Heter.	11.9	7.5–18.9	1.6	–
	Δα6 Homoz.	986.0	885–1,099	164.1	−0.991

Note

LC50: lethal concentration necessary to kill 50% of the insects; 95% CI: confidence interval at 95% level.

Flies homozygous for R81T showed moderate levels of resistance to imidacloprid, an N‐nitroguanidine neonicotinoid. The lethal concentration of imidacloprid necessary to kill 50% (LC50) of homozygous R81T flies was 32.6 times higher than that necessary to kill 50% of control flies with the same genetic background (LC50—resistance ratio (RR) of 32.6) (Table 1, Figure 3a). In contrast, only low levels of resistance were seen for the N‐cyanoamidine neonicotinoid, acetamiprid (LC50—RR of 6.5), the sulfoximine, sulfoxaflor (LC50—RR of 5.1) and the butanolide, and flupyradifurone (LC50—RR of 6.9) (Table 1, Figure 3b–d). When tested against spinosad, R81T homozygous flies were significantly more susceptible than control flies (LC50—RR of 0.4) (Table 1, Figure 3e). Flies that were heterozygous for R81T showed low but significant increases in resistance to imidacloprid (LC50—RR of 4.3), sulfoxaflor (LC50—RR of 2.3) and flupyradifurone (LC50—RR of 1.9) (Table 1, Figure 3a,c,d). The resistance caused by the R81T substitution was shown to be inherited as an incompletely recessive trait with dominance levels of −0.847, −0.892, −0.355 and −0.706 for imidacloprid, acetamiprid, sulfoxaflor and flupyradifurone, respectively (Table 1).

As has been reported previously (Perry et al., 2007; Zimmer et al., 2016), flies with a homozygous deletion of the *nAChR‐α6* gene (Δα6 flies) presented high levels of resistance to spinosad (LC50—RR of 164.1) (Table 1, Figure 3e). Also, in accordance with the previous studies, the mode of inheritance of the resistance was almost completely recessive (D = −0.991) (Table 1).

### 3.3 The R81T substitution in the nAChR_β1 subunit has a greater impact on the fitness of *D. melanogaster* than the knock‐out of the *nAChR_*α*6* gene

To investigate whether the resistance mutations confer any fitness costs to flies, strains carrying each of the two mutations, both in homozygosis and in heterozygosis, were assessed in comparison with a control strain for changes in fecundity, fertility, larval crawling, adult climbing and longevity.

**FIGURE 4:**
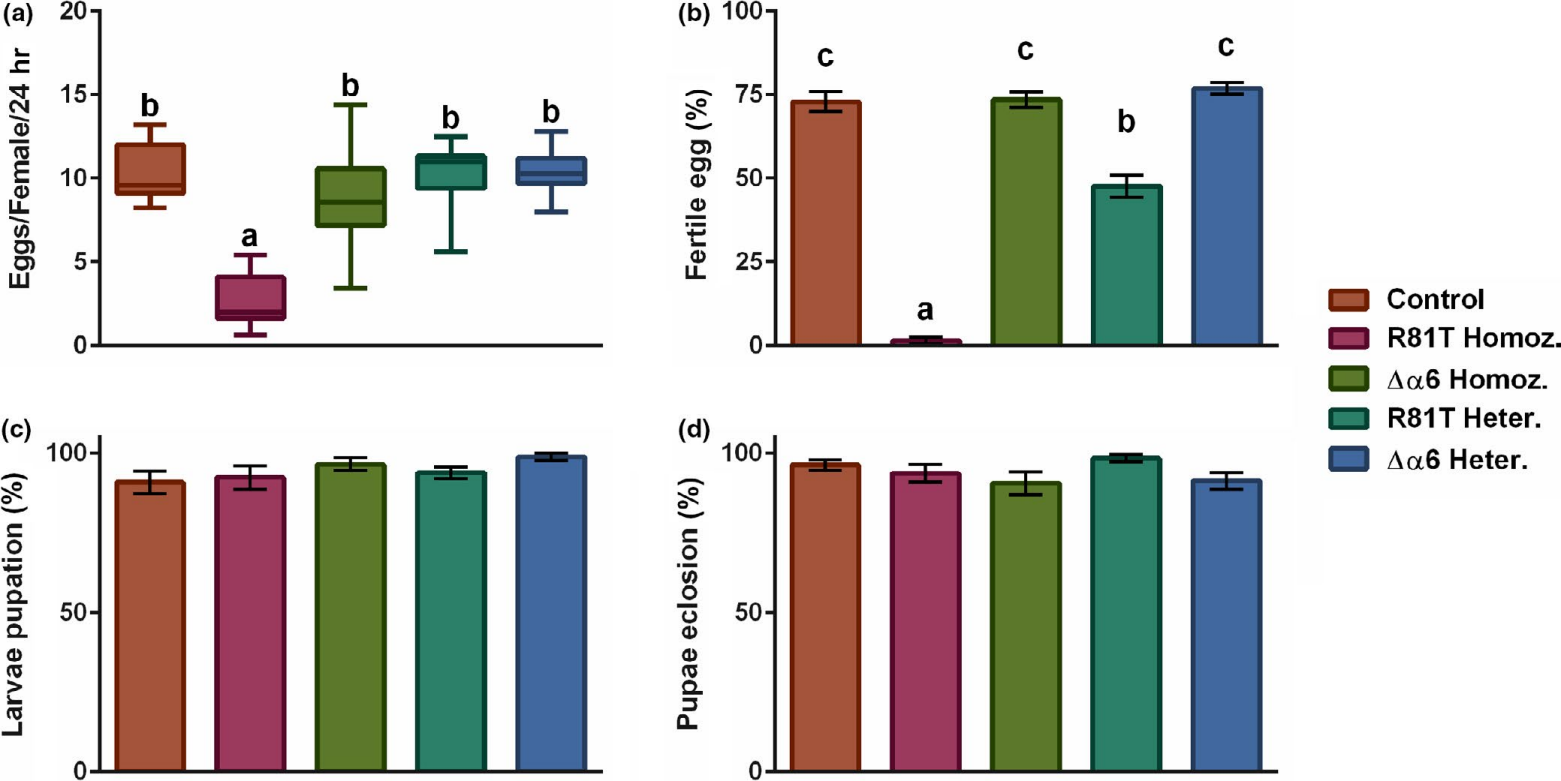
**Impact of nAChR resistance‐associated mutations on fecundity and fertility.** **(a)** The number of eggs laid by single young mated female flies in a period of 24 hr **(b)** the number of pupae that originated from those eggs **(c)** the proportion of larvae that pupated and **(d)** pupae that eclosed after being transferred to new food vials. Box plots represent the interquartile ranges and the median value (horizontal line within boxes). Whiskers indicate the most extreme data within a 1.5 interquartile range. Error bars represent S.E.M., and statistical significance is indicated by different letter codes above the bars (one‐way ANOVA and Bonferroni multiple comparison test)

Fecundity, measured by calculating the number of eggs produced by young mated female flies over a 24‐hr laying period, varied significantly between strains (ANOVA F_4,71_ = 42.43, *p* = < 0.001). R81T homozygous females laid significantly fewer eggs than the control strain (Bonferroni post hoc test, *p* = < 0.0001, Figure 4a). Whilst controls laid an average of 10.16 ± 0.67 (mean ± SE) eggs per female, the mean fecundity of R81T females was 2.59 ± 0.43. Δα6 homozygous, R81T heterozygous and Δα6 heterozygous presented a mean fecundity of 8.8 ± 0.58, 10.25 ± 0.43 and 10.4 ± 0.27, respectively (Figure 4a).

Fertility, measured by calculating the proportion of pupae generated from the laid eggs, also varied significantly between strains (ANOVA F_4,71_ = 148.5, p = < 0.0001). Both homozygous and heterozygous R81T females were significantly less fertile than the control (Bonferroni post hoc test, p = < 0.0001, Figure 4b). The percentage of fertile eggs laid by each strain was 72.88 ± 3.02% for controls, 1.52 ± 1.04% for R81T homozygous, 73.5 ± 2.19% for Δα6 homozygous, 47.71 ± 3.24% for R81T heterozygous and 76.9 ± 1.62% for Δα6 heterozygous (Figure 4b). To test whether the differences in fertility were caused by larval mortality, L1 larvae were collected and transferred to new vials to calculate the proportion that went on to pupate. No significant differences between fly strains were detected for pupation rates (Kruskal–Wallis test H = 5.529, 4 *df*, *p* = .2372) (Figure 4c) or for pupal eclosion rates (Kruskal–Wallis test H = 7.096, 4 *df*, *p* = .1309) (Figure 4d).

**FIGURE 5:**
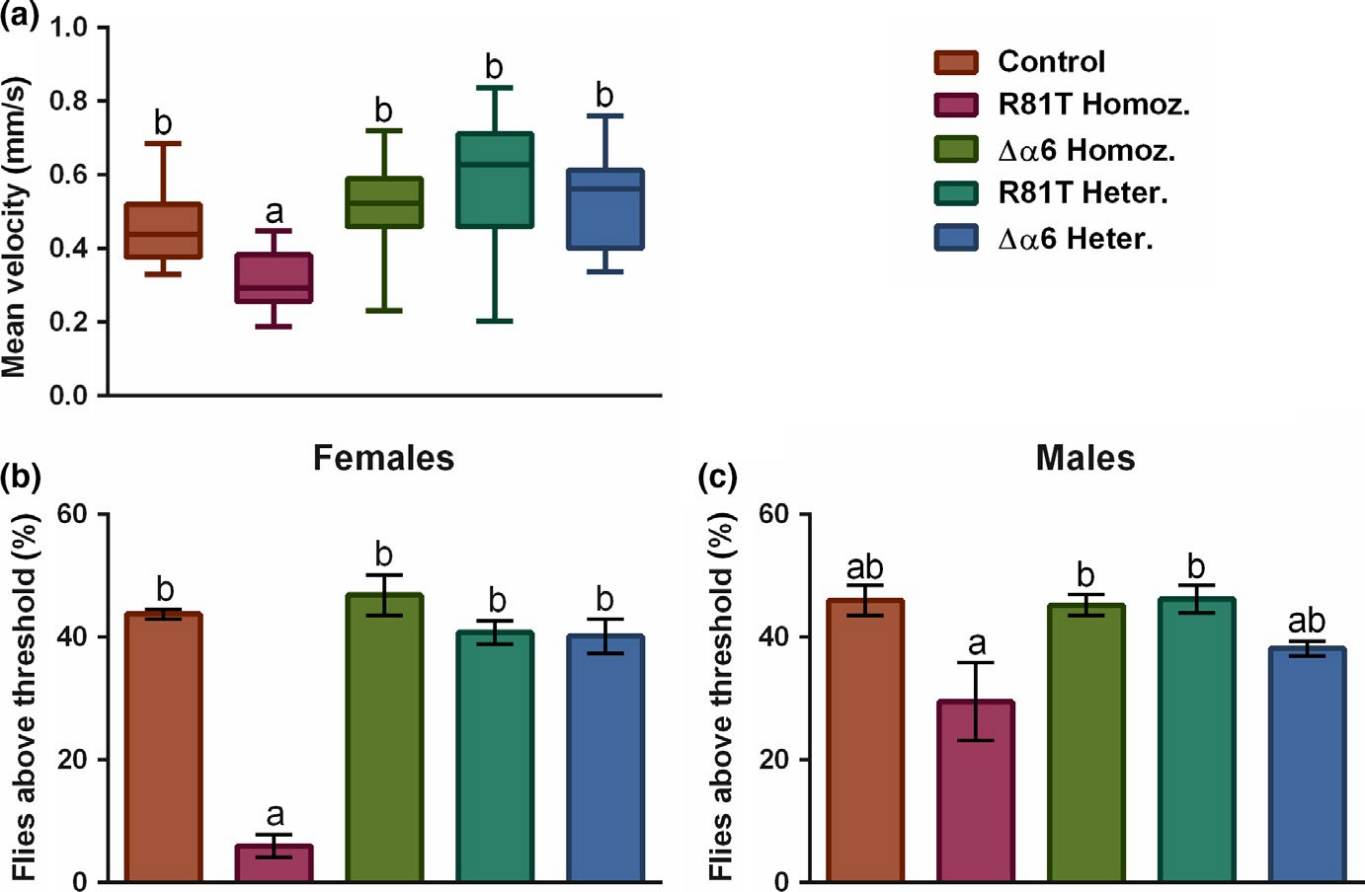
**Impact of nAChR resistance‐associated mutations on mobility.** **(a)** The median average speed of crawling larvae **(b)** the percentage of adult female and **(c)** male flies that climbed six centimetres or more in eight seconds after being struck to the bottom of a vial. Box plots represent the interquartile ranges and the median value (horizontal line within boxes). Whiskers indicate the most extreme data within a 1.5 interquartile range. Error bars represent S.E.M., and statistical significance is indicated by different letter codes above the bars (one‐way ANOVA and Bonferroni multiple comparison test)

Larval crawling performance, assessed by measuring the speed of second‐instar larvae moving on 2% agar/molasses plates, varied between strains (ANOVA F_4,79_ = 10.98, *p* < .0001). Larvae of the R81T homozygous strain were significantly slower than the control strain (Bonferroni post hoc test, p = < 0.05, Figure 5a). The mean velocity of R81T homozygous and control larvae was 0.310 ± 0.021 and 0.456 ± 0.025 (mm/sec ± SE), respectively. The other three strains, with mean velocities of 0.525 ± 0.030 mm/sec (Δα6 homozygous), 0.574 ± 0.039 mm/sec (R81T heterozygous) and 0.516 ± 0.031 mm/sec (Δα6 heterozygous), did not significantly differ from the control strain (Figure 5a).

Climbing performance, assessed by measuring the percentage of adult flies able to cross a mark at a height of 6 cm within 8 s of being forced to the bottom of a vial, varied significantly between strains (ANOVA F_4,30_ = 42.00, p = < 0.001). R81T homozygous females were significantly slower than the control strain (Bonferroni post hoc test, p = < 0.0001, Figure 5b). Whilst just 6.01 ± 1.85% of R81T homozygous female flies managed to climb past the mark, females of the other four strains had a success rate of > 40%, with 43.78 ± 0.81% of control females crossing the line over the same period of time (Figure 5a). These differences, however, were less clear for males (ANOVA F_4,30_ = 4.79, *p* =.004) (Figure 5b) where R81T homozygous flies were again the slowest, but the proportion that managed to cross the mark was not significantly different from that of controls (29.46 ± 6.34% versus 46.00 ± 2.41%) (Bonferroni post hoc test, p = > 0.05, Figure 5c). The deletion of the *nAChR_α*6 gene had no effect on climbing performance of males or females. The success rate observed for Δα6 homozygous flies was 46.86 ± 3.30% (females) and 45.23 ± 1.70% (males). For Δα6 heterozygous flies, these were 40.15 ± 2.76% (females) and 38.16 ± 1.26% (males) (Figure 5b‐c).

**FIGURE 6:**
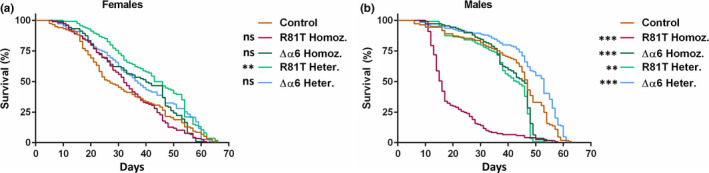
**Impact of nAChR resistance‐associated mutations on longevity.** **(a)** Lifespan of female and **(b)** male flies when maintained under optimal conditions at 25°C. Statistical significance was tested using the log‐rank (Mantel–Cox) test against the survival of control flies (**p < .001 and ***p < .0001). Statistics for survival curves are also summarized in Table S2

Longevity, or lifespan, measured by scoring mortality of flies over time, was impacted by both mutations (Figure 6a,b). R81T heterozygous females showed a significant increase in median lifespan when compared to control females (44.5 versus 27.0 days, *p* = .0002, log‐rank (Mantel–Cox) test), but the other strains did not differ from the controls (Figure 6a and Table S2). In contrast, males of all but the Δα6 heterozygous strain had a significant reduction in lifespan compared with the control males. The median lifespan of Δα6 heterozygous male flies was six days longer than that of control flies (53 versus 47 days, *p* = .0007). R81T homozygous males presented the shortest median lifespan of 16 days (Figure 6b and Table S2).

In summary, comparisons against control flies with the same genetic background showed that fecundity, fertility, crawling and climbing performances were all negatively impacted by the R81T substitution, but not by the deletion of the *nAChR_*α*6* gene. Longevity assays suggested that both mutations significantly reduce the lifespan of male flies but have no effect on the lifespan of females.

## 4 DISCUSSION

Resistance management strategies for pest insects are often based on rotating and/or combining insecticides with differing modes of action. It is assumed that, as resistance has a genetic basis, the frequency of an allele that confers resistance to one compound (with a presumed associated fitness cost) will decline when a second compound (or no compound) is used (see Cloyd, 2010). But is resistance always costly and how can this be best assessed? The answers to these questions are central to the formulation of effective pest management strategies (Ffrench‐Constant & Bass, 2017).

Previous studies aiming to gain an understanding of the relationship between pesticide resistance and fitness costs have mainly relied on time‐consuming and highly laborious methods to generate near‐isogenic lines (NILs). These methods involved multiple rounds of genotyping and/or selection‐driven backcrosses with the aim of replacing most of the genome of the resistant strain with the genome of the susceptible strain (Tsukamoto, 1983). Another drawback of NILs is that when alleles are in close proximity on a chromosome, they tend to be inherited together. If such alleles play an additive role in resistance, for example the kdr/ skdr mutations in the voltage‑gated sodium channel (Field,Davies, O’Reilly, Williamson, & Wallace, 2017), the dissection of the contribution of a particular allele towards resistance and any associated fitness costs becomes unfeasible. However, despite the technical and genetic barriers, the generation of NILs has been successfully used to study insecticide resistance and fitness in a number of arthropod species of economic importance including the Australian sheep blowfly, *Lucilia cuprina* (McKenzie, Whitten, & Adena, 1982), the fall armyworm, *Spodoptera frugiperda* (Horikoshi et al., 2016), the diamondback moth, *P. xylostella* (Zhu et al., 2016), the western flower thrip, *Frankliniella occidentalis* (Li et al., 2017), the common housefly, *Musca domestica* (Azhar, Khan, & Khan, 2018), and the two‐spotted spider mite, *Tetranychus urticae* (Bajda et al., 2018).

Recent advances in genome‐editing technologies have allowed precision‐targeted modifications in the genome of eukaryotes (Gaj, Gersbach, & Barbas, 2013). This has proven particularly useful to validate target‐site resistance mutations (reviewed in Homem & Davies, 2018) and, as presented in this study, to test whether these mutations carry any fitness penalties. By introducing resistance mutations in the same genetic background, comparisons between resistant and susceptible strains can easily and reliably be made. Not surprisingly, the model organism *D. melanogaster* has pioneered these studies. In fact, the first two target‐site resistance mutations edited in the genome of an insect were introduced in the *nAChR_*α*6* gene of *D. melanogaster* using CRISPR/Cas9 (Somers et al., 2015; Zimmer et al., 2016).

Early site‐directed mutagenesis studies using recombinant chicken nAChR proteins suggested that the low affinity of the neonicotinoid imidacloprid towards the vertebrate receptor was due to the presence of a threonine at position 77 (T77) and a glutamic acid at position 79 (E79) of the β2 subunit (Shimomura et al., 2006). Amino acid replacements that mimic the insect receptor (T77R and E79V) drastically increased the affinity of the receptor to imidacloprid. Based on computational modelling, the study also suggested that the higher affinity towards the mimicked insect receptors was mainly due to the interaction of the nitro group of imidacloprid with the basic residues at position 77. It was therefore interesting when analogous R‐to‐T substitutions were later found in the *nAChR_β1* subunit of neonicotinoid resistant populations of two aphid species, *M. persicae* (Bass et al., 2011) and *Aphis gossypii* (Koo, An, Park, Kim, & Kim, 2014; Shi et al., 2012), suggesting a role for this substitution in resistance to neonicotinoid insecticides.

The present study confirms that the R81T substitution causes resistance to neonicotinoid insecticides and in accordance with previous studies, there is a greater effect on the resistance to imidacloprid, a N‐nitroguanidine neonicotinoid, compared with acetamiprid, a N‐cyanoamidine neonicotinoid and sulfoxaflor, a sulfoxamine insecticide (Hirata, Jouraku, Kuwazaki, Kanazawa, & Iwasa, 2017; Wang, Watson, Loso, & Sparks, 2016). The results also suggest that the R81T substitution causes low levels of cross‐resistance to flupyradifurone, a relatively new butenolide insecticide that has been proposed as an alternative to neonicotinoids (Nauen et al., 2015). However, differences in the magnitude of the resistance ratios observed for aphids compared to our genome‐edited flies suggest that R81T is not the only mechanism of resistance in the *M. persicae* and *A. gossypii* strains. This is supported by the fact that, at least in *M. persicae*, metabolic resistance mediated by the cytochrome P450 CYP6CY3 plays a major role in neonicotinoid resistance (Bass et al., 2013; Puinean et al., 2010).

In contrast to neonicotinoids, target‐site mutations that confer resistance to spinosyns have already been identified in many insect pest species (Bao et al., 2014; Baxter et al., 2010; Berger et al., 2016; Hsu et al., 2012; Puinean, Lansdell, Collins, Bielza, & Millar, 2013; Silva et al., 2016; Wan et al., 2018; Wang, Wang, et al., 2016), the first being a null mutation in the *nAChR_α6* gene of *D. melanogaster* (Perry et al., 2007). The high degree of conservation of nAChR subunits across insect species, combined with no obvious fitness costs associated with this mutation in *D. melanogaster* (no differences were observed in survival from first‐instar larval stage to adulthood), led Perry et al. (Perry et al., 2007) to speculate that spinosad resistance in field populations could be caused by loss‐of‐function mutations in *nAChR_α6* orthologs. This was later confirmed in crop pests including the diamondback moth, *P. xylostella* (Baxter et al., 2010; Wang, Wang et al., 2016), the oriental fruit fly, *Bactrocera dorsalis* (Hsu et al., 2012), the tomato leaf miner, *Tuta absoluta* (Berger et al., 2016), and western flower thrips, *Frankliniella occidentalis* (Wan et al., 2018).

In line with data presented here, we speculate that fitness costs (or lack of) may at least partially explain the difference between the rapid and widespread evolution of target‐site resistance to spinosad compared with the slow and restricted evolution of target‐site resistance to neonicotinoids. Our data show that the R81T substitution confers a significant fitness cost to homozygous but not heterozygous *D. melanogaster* strains, with homozygous female flies producing fewer and less fertile eggs. R81T homozygous larvae were also shown to be slower at crawling than the other strains, whilst adults were less responsive in climbing assays. Furthermore, R81T homozygous and heterozygous male flies had a shorter median lifespan when compared to male control flies. Altogether, these results suggest that a strong fitness cost is associated with the R81T substitution in *D. melanogaster*. In contrast, flies resistant to spinosad, which had almost their entire *nAChR_α6* gene deleted, performed similarly to the controls in all but the longevity assay, where both homozygous and heterozygous males presented shorter median lifespans when compared to male control flies. These results suggest a very low fitness cost associated with target‐site resistance to spinosad and are in accordance with what has been observed in other spinosad‐resistant *D. melanogaster* strains (Perry et al., 2007; Zimmer et al., 2016).

But why does a single amino acid change in the β1 subunit seem to be much more detrimental to flies than the deletion of the whole α6 subunit? To answer this question, one must first understand how the receptor assembles and functions. nAChRs are formed of five subunits, each presenting a di‐cysteine loop (Cys‐loop) in the N‐terminal, extracellular domain. The subunits are classified into α and non‐α types according to the presence (α subunits) or absence (non‐α subunits) of vicinal cysteine residues in the loop C. The combination of different subunits forms multiple functional heteropentamers or homopentamers with distinct functional and pharmacological profiles. The interface between two subunits presents six separate loops (A–F) that make up the binding pocket of ACh, the molecule that mediates cholinergic neurotransmission (Matsuda, Shimomura, Ihara, Akamatsu, & Sattelle, 2005). Competitive inhibition assays using ACh and [^3^H] imidacloprid suggested that imidacloprid and ACh bind to the same pocket in the nAChR (Liu, Latli, & Casida, 1995). This was later supported by crystallography studies using *Lymnaea stagnalis* acetylcholine‐binding protein (Ls‐AChBP), a protein structurally similar to the extracellular domain of nAChRs and presenting all the six A–F loops that make up the binding site of ACh (Ihara et al., 2008). Site‐directed mutagenesis of the chicken α7 nAChR subunit expressed in *Xenopus laevis* oocytes showed that mutations of the residue at position 79, equivalent to position 81 in *M. persicae* β1 subunit, not only interfered with the affinity towards neonicotinoids but also, to a lesser extent, affected the interaction with ACh (Shimomura et al., 2002). Therefore, the phenotypes observed in *D. melanogaster* carrying the R81T substitution could be related to an alteration in the responsiveness of the receptor towards ACh. Such alterations are likely to disturb cholinergic neurotransmission and impair biological functions. The lack of fitness costs associated with the deletion of the *nAChR_α6* gene is probably due to the redundancy of this subunit for the formation of functional receptors. As presented here and by others, this gene is not essential for the viability of insects (Baxter et al., 2010; Hsu et al., 2012; Perry et al., 2007; Rinkevich et al., 2010; Zimmer et al., 2016). Conversely, this is unlikely to be true for the *nAChR_β1* gene in which a loss‐of‐function mutation has never been reported. In addition, spinosad has been shown to bind to the C‐terminal region of the α6 subunit (Somers et al., 2015), away from the ACh binding pocket. Therefore, even spinosad resistance mutations that do not result in loss of function (Puinean, Lansdell, et al., 2013; Silva et al., 2016; Somers et al., 2015) are less likely to interfere with the affinity of the receptor towards ACh.

Altogether, the results presented here demonstrate that an R81T substitution in the nAChR_β1 subunit of *D. melanogaster* confers a moderate level of resistance to neonicotinoids (and other insecticides that target this receptor) and carries a significant fitness cost to the insect. Combined with the fact that field‐evolved (R81T‐associated) resistance to neonicotinoids has only been detected in two aphid species, despite the long term and widespread use of these insecticides, this suggest that fitness costs associated with such mutations in the nAChR_β1 subunit may have precluded target‐site resistance from evolving in other insect species. By contrast, the apparent low fitness costs combined with the high levels of insecticide resistance caused by loss‐of‐function mutations in the nAChR_α6 subunit might have accelerated the evolution of target‐site resistance to spinosad in multiple insect species. It is important to highlight here, however, that a degree of caution should be applied when extrapolating experimental findings across species as conflicting results have previously been reported using different methodologies. For example, chitin synthases are enzymes involved in chitin biosynthesis and are the target of etoxazole, benzoylurea and buprofezin insecticides/acaricides. Resistance to these compounds has been attributed to mutations (I1017F, I1042M) in the equivalent position on the chitin synthase (*CHS1*) gene of *T. urticae* (Van Leeuwen et al., 2012) and *P. xylostella* (Douris et al., 2016). Douris and co‐workers (Douris et al., 2016) used CRISPR/Cas9 to introduce the equivalent mutations (I1056M/F) in the ortholog *CHS1* gene of *D. melanogaster* and showed that they indeed confer strong levels of resistance against CHS inhibitors. The authors also reported that the mutations had no impact on the fitness of these modified flies in comparison with a control of identical genetic background. However, a latter study using *T. urticae* NILs suggested that the I1017F mutation is associated with a significant fitness cost in spider mites (Bajda et al., 2018). Therefore, to avoid any possible misinterpretations, trade‐offs between resistance and fitness should ideally always be assessed in the organism in which resistance has evolved. However, this is not always practical; for example, several species of aphid predominantly present a parthenogenetic life cycle and this makes genetic crosses highly challenging and the generation of NILs impractical. Furthermore, despite a recent report on the first successful generation of a genome‐edited aphid strain using CRISPR/Cas9 (Le Trionnaire et al., 2019), genome editing in aphids remains extremely challenging and laborious. Due to such technical challenges, it has not yet been possible to assess the fitness cost of the R81T substitution directly in aphids. Thus, alternative strategies such as the use of *D. melanogaster* as a model system, although not ideal, are of great value in shedding light on the potential impact of such resistance mutations on fitness. We would also emphasize that since environmental factors can play a significant role in influencing fitness costs associated with resistance, what is observed in a controlled environment laboratory study may not truly reflect what happens in the field. In order to further validate our hypothesis that fitness costs (or the lack of) could play an important role in shaping the spatial and temporal development of target‐site resistance against neonicotinoids and spinosyns, we would need to introduce these mutations into the genome of other insect species. Ideally, these modified strains should then be tested under semi‐realistic field conditions and in competition with insects sharing the same genetic background. Fortunately, recent advances in genetic transformation and genome editing in nonmodel insects (rather than our current reliance on Drosophila) will soon allow scientist to validate these hypotheses in the actual pest insect under semi‐realistic field conditions.

## AUTHOR CONTRIBUTIONS

R.A.H., M.W., L.M.F. and T.G.E.D. conceived the project and designed the experiments. R.A.H., B.B., E.R. and Y. T. performed the experiments. R.A.H. analysed the data. R.A.H. and T.G.E.D wrote the manuscript with feedback from all authors.

## Supporting information

FIGURE S1: Click here for additional data file.

TABLE S1: Click here for additional data file.

TABLE S2: Click here for additional data file.

## Data Availability

Raw data that support the findings of this study are openly available in Dryad at http://doi:10.5061/dryad.v9s4mw6sc. Primer sequences are provided in Supporting Information. Flies strains will be made available upon request.
