# Supplementary figures and images for: Evolutionary trade‐offs of insecticide resistance — The fitness costs associated with target‐site mutations in the nAChR of Drosophila melanogaster
Source: Mol Ecol. 2020 Jun 22;29(14):2661–75. doi: 10.1111/mec.15503 (PMC7496652; doi:10.1111/mec.15503)

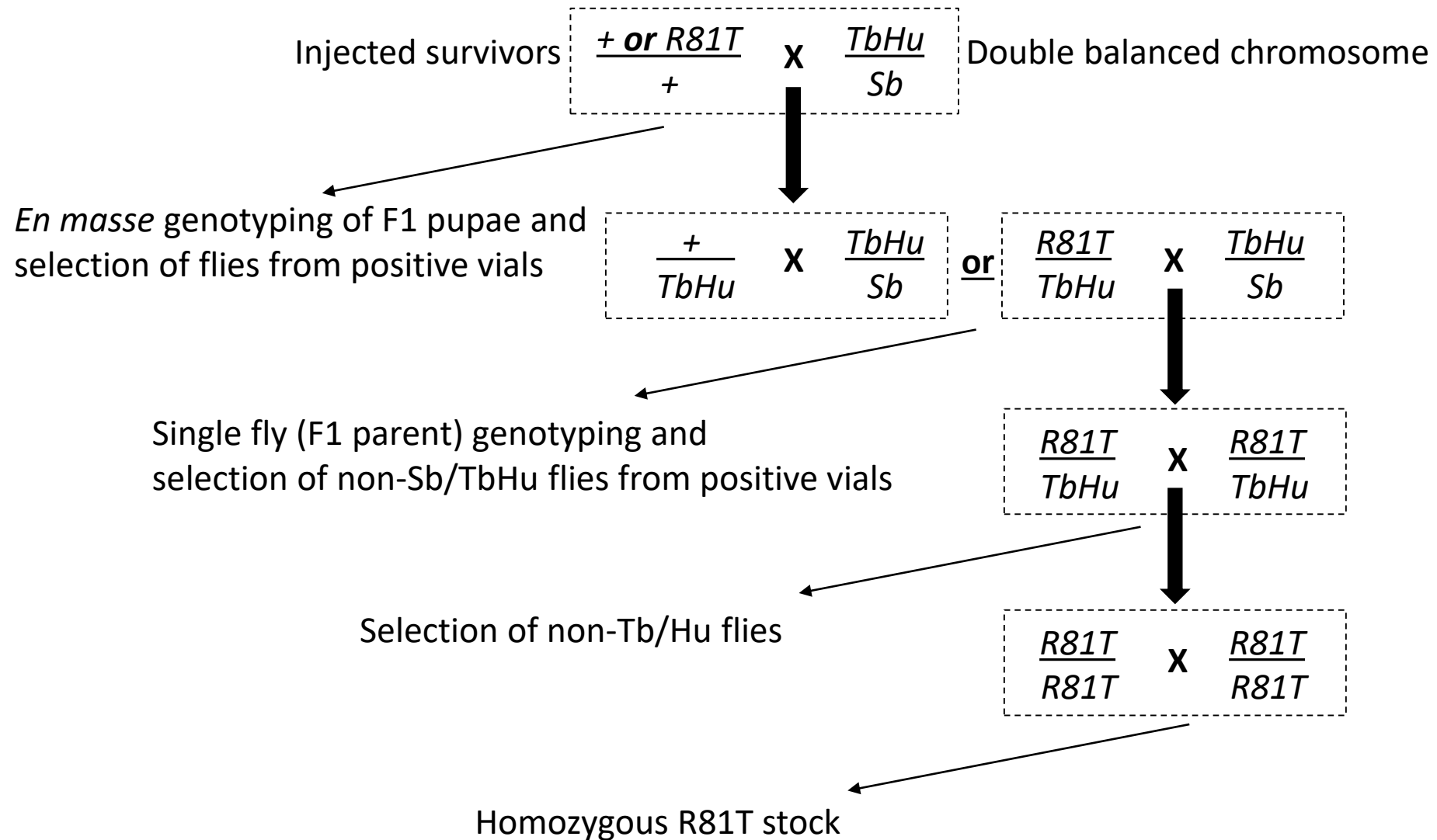

Supplement: Supplementary file 1 — FIGURE S1: [file MEC-29-2661-s001.pdf]
