# Supplementary material for: Evolutionary trade‐offs of insecticide resistance — The fitness costs associated with target‐site mutations in the nAChR of Drosophila melanogaster
Source: Mol Ecol. 2020 Jun 22;29(14):2661–75. doi: 10.1111/mec.15503 (PMC7496652; doi:10.1111/mec.15503)
Supplement: Supplementary file 2 — TABLE S1: [file MEC-29-2661-s002.docx]

| ***Supplementary Table 1:* List of primers used in the study** | | |
| --- | --- | --- |
| **Primer name** | **Purpose** | **Sequence (5’-3’)** |
| A6_Seq_5’_gRNA_F | Sequencing gRNA target site on 5’ end of the nAChR_α6 gene | AATTTTCTTTGACTTTTCTT |
| A6_Seq_5’_gRNA_R |  | TTGAGTCAAATTGTGGAT |
| A6_Seq_3’_gRNA_F | Sequencing gRNA target site on 3’ end of the nAChR_α6 gene | AAGAGTGTAGAAAATGGTTT |
| A6_Seq_3’_gRNA_R |  | AGATTTGATATGAGACGATT |
| A6_DsRed_int_5`_F | Genotyping nAChR_α6 knock-out/ DsRed knock-in | ATACGAAGTTATCTACGC |
| A6_DsRed_int_5`_R |  | TGATAAAAATGATAAGTGTG |
| A6_DsRed_int_3`_F | Genotyping nAChR_α6 knock-out/ DsRed knock-in | TTTTGCTTTTTGACTTA |
| A6_DsRed_int_3`_R |  | GAGCACTAGTATAACTTCGT |
| A6_RT-PCR_F | Validating nAChR_α6 knock-out | CCGCGCCGTAATAAATTGCA |
| A6_RT-PCR_R |  | GCATCATGTTCATGGTCGCC |
| R81T_RT-PCR_F | Validating R81T knock-in | CCGCGCCGTAATAAATTGCA |
| R81T_RT-PCR_R |  | GCATCATGTTCATGGTCGCC |
| B1_Seq_HA_Left_F | Sequencing nAChR_β1 target region and genotyping R81T integration | GGCGCATTCAACGTTTCAGT |
| B1_Seq_HA_Left_R |  | ATGGTGGTGGTTTGGTGGTT |
| B1_Seq_HA_Right_F | Sequencing nAChR_β1 target region | CGTACCCGTACTTCGTACCG |
| B1_Seq_HA_Right_R |  | AGGTCACATCGATGGTGCAG |
| attP2_BglII_F | Restriction ligation cloning of second attP site into pDsRed-attP to generate pDsRed-2attP | TGAAGATCTGTAGTGCCCCAACTGGG |
| attP2_SpeI_R |  | GCCACTAGTATAACTTCGTATAGCATACATTATACG |
| A6_5'_HA_SphI_F | Restriction ligation cloning nAChR_α6 5’ homology arm into pDsRed-2attP | GCATGCCCAAGGCAGGCGAATGGC |
| A6_5'_HA_EcoRI_R |  | GAATTCATCGCTGCAGCTGTCGCTGTCTC |
| A6_3'_HA_XhoI_F | Restriction ligation cloning nAChR_α6 3’ homology arm into pDsRed-2attP | CTCGAGTAACGGTTTAATAATTATGCAATAAG |
| A6_3'_HA_BglII_R |  | AGATCTCGGAGGGTGTGTGCATTTT |
| A6_3’_gRNA_pCDF4_F | Gibson assembly of nAChR_α6 3’ and 5’ gRNA sequences into pCFD4 | TATATAGGAAAGATATCCGGGTGAACTTCGCTAACGGTACACACACGCCAGTTTTAGAGCTAGAAATAGCAAG |
| A6_5’_gRNA_pCDF4_R |  | ATTTTAACTTGCTATTTCTAGCTCTAAAACCCGCAGCTCTCGCTCTCTATCGACGTTAAATTGAAAATAGGTC |
| B1_left_gRNA_pCDF4_F | Gibson assembly of nAChR_β1 left and right gRNA sequences into pCFD4 | TATATAGGAAAGATATCCGGGTGAACTTCGTGATTGTGATCAGATGGTGGGTTTTAGAGCTAGAAATAGCAAG |
| B1_right_gRNA_pCDF4_R |  | ATTTTAACTTGCTATTTCTAGCTCTAAAACCCGTAGTCGGCCTCATCCCACGACGTTAAATTGAAAATAGGTC |
| RpL32_F | Housekeeping genes used as control in RT-PCRs | GCGCTTGTTCGATCCGTAAC |
| RpL32_R |  | GCCCAAGGGTATCGACAACA |
