# Supplementary material for: Evolutionary trade‐offs of insecticide resistance — The fitness costs associated with target‐site mutations in the nAChR of Drosophila melanogaster
Source: Mol Ecol. 2020 Jun 22;29(14):2661–75. doi: 10.1111/mec.15503 (PMC7496652; doi:10.1111/mec.15503)
Supplement: Supplementary file 3 — TABLE S2: [file MEC-29-2661-s003.docx]

***Supplementary Table 2:* Results of Log-rank (Mantel–Cox) tests from survival data**

| **Females** | **n** | **Median (days)** | **X^2^** | **Log-rank (vs Control)** |
| --- | --- | --- | --- | --- |
| **Control** | 117 | 27 | - | - |
| **R81T Homoz.** | 118 | 32 | 0.4895 | p=0.4841 |
| **Δα6 Homoz.** | 103 | 40 | 0.4814 | p=0.4878 |
| **R81T Heter.** | 114 | 44.5 | 13.64 | p=0.0002 |
| **Δα6 Heter.** | 111 | 37 | 3.368 | p=0.0665 |

| **Males** | **n** | **Median (days)** | **X^2^** | **Log-rank (vs Control)** |
| --- | --- | --- | --- | --- |
| **Control** | 109 | 47 | - | - |
| **R81T Homoz.** | 121 | 16 | 112.9 | p<0.0001 |
| **Δα6 Homoz.** | 109 | 46 | 16.06 | p<0.0001 |
| **R81T Heter.** | 119 | 43 | 25.63 | p<0.0001 |
| **Δα6 Heter.** | 124 | 53 | 11.48 | p=0.0007 |
